# The Impact of Hypermobile “Ehlers-Danlos Syndrome” and Hypermobile Spectrum Disorder on Interpersonal Interactions and Relationships

**DOI:** 10.3389/fresc.2022.832806

**Published:** 2022-04-11

**Authors:** Stijn De Baets, Marieke De Temmerman, Patrick Calders, Fransiska Malfait, Geert Van Hove, Guy Vanderstraeten, Inge De Wandele, Dominique Van de Velde

**Affiliations:** ^1^Occupational Therapy Research Group, Department of Rehabilitation Sciences, Faculty of Medicine and Health Sciences, Ghent University, Ghent, Belgium; ^2^Department of Rehabilitation Sciences, Faculty of Medicine and Health Sciences, Ghent University, Ghent, Belgium; ^3^Centre for Medical Genetics, Ghent University Hospital, Ghent, Belgium; ^4^Department of Special Needs Education, Faculty of Psychology and Educational Sciences, Ghent University, Ghent, Belgium; ^5^Department of Physical and Rehabilitation Medicine, Ghent University Hospital, Ghent, Belgium

**Keywords:** hypermobile Ehlers-Danlos Syndrome, Ehlers-Danlos Syndrome, Hypermobility Spectrum Disorder, interpersonal interactions, participation

## Abstract

**Background:**

People with Ehlers-Danlos Syndromes and Hypermobility Spectrum Disorders are hampered in their social participation, especially in the social relationships they have.

**Objective:**

The aim of this study is to research the impact of hypermobile Ehlers-Danlos Syndrome (hEDS) and Hypermobile Spectrum Disorders (HSD) on interpersonal interactions and relationships.

**Methods:**

A phenomenological hermeneutic study was performed. Semi-structured interviews were used to explore the experiences of 11 participants.

**Results:**

Four themes emerged from the data analysis. (1) people with hEDS or HSD can no longer do what they want to do and that affects their identity, (2) people with hEDS or HSD have to find a balance in the amount of activities they participate in, (3) having hEDS or HSD influences how to ask for, accept and give help, and (4) Relationships are affected in persons with hEDS or HSD. As well as changes in the social network, different types of relationships are influenced by the disease, including relationship with their partner, their children, their friends, strangers, fellow-sufferers and health care professionals.

## Introduction

Ehlers-Danlos Syndromes (EDS) comprise a clinically and genetically heterogeneous group of hereditary connective tissue disorders that are generally caused by a collagen synthesis defect (1). EDS is characterized by three main characteristics: skin hyper extensibility, soft tissue fragility and joint hypermobility. Hypermobile Ehlers-Danlos Syndrome (hEDS) is the most common subtype (2). The genetic defects underlying this type are unknown, so no genetic testing is currently available (3). In 2017, the American Journal of Medical Genetics published an updated EDS classification (4). For a diagnosis of hEDS, the presence of symptomatic generalized joint hypermobility is required, in combination with multiple signs and symptoms that argue for an underlying heritable connective tissue disorder. These include skin marks, signs of soft tissue fragility, and several morphological features. The third criterion consists of a thorough exclusion of other heritable connective tissue disorders, musculoskeletal pathologies and other heritable syndromes. Besides these diagnostic signs, patients report multisystemic symptoms that decrease their quality of life, including recurrent joint dislocations, muscle weakness, severe chronic pain, fatigue, gastrointestinal problems and problems with blood pressure regulation (5, 6). hEDS is a complex, chronic disorder leading to disabilities that limit daily activities (2). Several studies reported that hEDS causes physical and psychosocial impairments (7, 8).

“Living with limitations” is a central theme for patients with hEDS (9, 10). In individuals with symptomatic joint hypermobility (JH) who do not meet the criteria for hEDS, the term Hypermobility Spectrum Disorders (HSD) is proposed (11). Castori et al. (11) define HSD as a group of clinically relevant conditions related to JH, intending it as a descriptive and exclusion diagnosis. HSD, just like hEDS, has a significant impact on quality of life.

The clinical manifestations of HSD and hEDS can be equal in severity, so both need similar management (4). In the past, patients' experiences of living with hEDS have been neglected (12), but recent studies have started to highlight the need for a biopsychosocial approach (13–20). Tinkle et al. (21) suggest that the approach for people with hEDS or HSD should be holistic, focusing on the complications, quality of life and functionality, as well as the psychological aspects (21). Social participation has the potential to strengthen an individual's social relations (22). Pietromonaco and Collins (23) note that social connections shape biological responses and behaviors that are consequential for health in three key interpersonal processes: social support in the context of stressful life events, social support in the context of exploration, striving toward goal and positive life events, and intimacy, affection and love (23). Social connections can help individuals sustain a positive affective balance, facilitate emotion regulation, and build resilience (24). Support for exploration and intimate interactions can also increase participation in leisure activities (23, 25).

Bennet et al. (17) point to some themes that enrich the body of knowledge regarding the psychosocial impact of EDS (17, 18). Several relevant topics were identified. (1) Restrictions imposed by JHS/EDS-HT, focusing mainly on activities of daily life that are restricted due to dislocations, fatigue and pain, mobility restrictions and difficulties with intimate relationships. (2) Healthcare limitations, with a focus on the limitations of current treatment. (3) Social stigma is of major importance. Participants indicate having difficulties with keeping up with friends, family and colleagues, leading to feelings of frustration and anger. Also judgement of others due to the invisible nature of the disease is evident. (4) Fear of the unknown impacts the insecurity felt when making plans in different life areas. (5) The way of coping with EDS influences social relationships. (6) A lack of professional understanding. (7) Living a restricted life due to the fluctuating nature, and fear of future injuries, which on its turn leads to limited social participation, (8)Trying to keep up, in order to not “ruin” the perception of others of them. (9) Gaining control of their lives also seems to be of great importance in matters such as “redefining normality” (17, 18).

It is obvious that (social) participation and the impact of interpersonal interactions on health and wellbeing should be considered as important subjects in persons with EDS and HSD. Previous research has not focused on the impact of the disease on identity and interpersonal relations. Since social needs are a major concern for people with a disabling condition (26) and since it is important to gain insight into the extent to which people are hampered in their social participation, this paper aims to find an answer to the research question: “What is the perceived impact of hEDS and HSD on identity and interpersonal interactions?”

## Materials and Methods

### Study Design

This study used a qualitative research design with a phenomenological hermeneutic method, based on the theory of Lindseth and Norberg (27), taking into account the hermeneutic principles of Gadamer et al. (28–30), in order to get insight in the lived experiences of persons with EDS and HSD. This design involves three phases. The naïve understanding is followed by a structural analysis. The final phase contains a comprehensive understanding. All the participants signed an informed consent form. The study was approved by the Ethical Committee of Ghent University (B670201734047).

### Sampling

Criterion sampling as a purposive sampling method was used to select the participants (31). The participants were approached via an advertisement on the Facebook^®^ page of the Flemish EDS Association based on the following criteria: diagnosed with hEDS or HSD at the Medical Genetics Centre, Ghent University Hospital, Belgium, Dutch-speaking, living in Belgium, and 18 years or older. They were asked to send an email to the first author of the study if they were willing to participate. In a second phase, the diagnosis was confirmed, the entire research plan was clarified and the participants were asked to sign an informed consent form.

### Data Collection

Data were collected through individual semi-structured in-depth interviews at the homes of the participants in their native language, Dutch. Other family members did not participate in the conversation. A topic list was used (see Table 1) and the interviews were audiotaped. The questions were asked by using “open ended” questions. All interviewers had previous experience in conducting qualitative interviews. Two junior researchers under the supervision of a senior researcher conducted the interviews. The interviewers were not involved in the (para)medical care of the participants. The participants were asked to talk about the impact of the disease on their interpersonal interactions and relationships throughout daily life. Further questions were used as prompts to get more details. The interviews were recorded. The quality of the final manuscript was assessed using the Standards for Reporting Qualitative Research (SRQR) (32), a 21-item checklist for reporting qualitative research.

**Table 1:** Semi-structured interview guide.

**General information about the participant**		
**Name:**		
**Age:**		
**Profession:**		
**Place of residence:**		
**Diagnosis:**		
**Family situation:**		
Topic List		
The topic list was drawn up to prepare more specific questions which can be asked during the interviews. Since the interviews will be semi-structured, the questions are only a guideline and the topic list can help the researcher think about other questions during the interview, as long as they line up with the topics mentioned in this list. This to make sure that only topics that reflect on the research question will be discussed.		
**Topic**	**Subtopic**	**More specific**
Activities	Meaningful activities	Work/education
		Leisure time
		Selfcare
	Performance of activities	General performance
		Specific problems
EDS	Experiences	Influence of the diagnosis
		Self-image
	Limitations	Physical
		Social
	Benefits	Physical
		Social
Relationships	Formal relationships	Acceptance of others
	Informal relationships	Making friends
		Social activities
		Intimate relationships
	Communication	Contact with others
	Help	Asking help
		Accepting help
		Giving help

### Data Analysis

The audiotapes of the interviews were transcribed verbatim. The transcripts were analyzed by the first and second authors, with peer-debriefing with the senior researchers in order to ensure that the analysis remained true to the hermeneutic principles. One of the core elements that were taken into account was the “pre-understandings”. The researchers involved in the research process and analysis were not involved in the (para)medical care of the participants, although they are healthcare researchers (33). The research was an inductive and iterative back-and-forth process of data gathering and analysis in order to explore the individual perception of a participant and is subsequently interpreted by using existing theories and knowledge. Within the phenomenological–hermeneutical method, the easiest and most natural way of gathering information is to narrate from lived experiences, an approach that requires cooperation between the interviewer and the interviewee. This method was used so that the questions of the subsequent interview could be adjusted before further data were collected and analyzed (34). During this methodological procedure, three steps should be followed. As a first step, the transcripts were read in their entirety to achieve a naïve understanding and an overall picture of the data. The texts are read several times in order to understand their full meaning. This step is the first analysis. The naïve understanding is a baseline for the structural analysis. The structural analysis is intended to validate the naïve understanding. Connections and patterns in the data were uncovered by dividing the data into meaning units, condensed meanings, sub-themes and themes. The structural analysis delivers the method of interpretation as a way to formulate themes. A theme can be seen as a thread of meaning that penetrates parts of the text. Themes are described as “condensed descriptions” that are formulated in a way that discloses meaning(s). The text is read and divided into meaning units. Next, the themes are condensed, which means that the essential meaning is expressed in everyday words. Similar meaning units are further condensed into meaningful topics, which finally contain the themes. The third phase consisted of identifying relationships between the various themes by means of a comprehensive understanding. To achieve a comprehensive understanding of the data, the findings were re-contextualized by once again reading the texts as a whole, taking into consideration (a) the research question, (b) the pre-understanding, (c) the naïve understanding and (d) the results of the structural analysis. The gathered information can be interpreted in the light of the already existing literature (27). The themes and quotations were translated into English by a native speaker, by means of a back and forth translation process.

## Results

### Participants

Twelve participants were included, but one participant did not meet the criteria for purposive sampling due to an updated medical diagnosis. Based on this, that person was excluded. An overview of the participants is given in Table 2.

**Table 2:** Sample characteristics (*n* = 11).

**Participant**	**1**	**2**	**3**	**4**	**5**	**6**	**7**	**8**	**9**	**10**	**11**
**Age**	21	54	47	51	35	36	35	42	32	33	32
**Gender**
Female	♦	♦	♦	♦	♦	♦		♦	♦	♦	♦
Male							♦				
**Diagnosis**
hEDS		♦	♦	♦		♦	♦	♦		♦	
HSD	♦				♦				♦		♦
Since	2017	2017	2002	2011	2017	2017	2012	2017	2017	2017	2017
**Current occupation**
Paid employment		♦	♦				♦	♦	♦	♦	♦
Non-paid work				♦							
Student	♦										
Disability allowance				♦	♦	♦					
**Civil status**
Single	♦		♦								
In a relationship		♦		♦	♦	♦	♦	♦	♦	♦	♦
With children		♦		♦	♦		♦	♦	♦	♦	
**Housing condition**
Living alone			♦					♦		♦	
Living with parents	♦										
Living with partner		♦		♦	♦	♦	♦		♦		♦

An overview of the duration of each interview is shown in Table 3.

**Table 3:** Duration of the interviews in minutes.

**Participant**	**1**	**2**	**3**	**4**	**5**	**6**	**7**	**8**	**9**	**10**	**11**	
Minutes	42	58	98	80	59	94	94	74	59	43	47	
Total = 835 min

### Phase 1, Naïve Understanding

The narratives showed that persons with hEDS and HSD experience limits in social relations and interactions over the long term. It is difficult for some people to actively take part in society in the way they used to. The amount and the quality of interpersonal relations has changed. So, having hEDS or HSD significantly impacts a person's quality of life.

### Phase 2, Structural Analysis

A structural analysis was applied to examine how the participants experience their interpersonal interactions and relationships. This resulted in four themes (Table 4).

**Table 4:** Themes.

1. Relationships are affected in persons with hEDS or HSD
2. Having hEDS or HSD influences how to ask for, accept and give help
3. People with hEDS or HSD can no longer do what they want to do and that affects their identity
4. People with hEDS or HSD must find a balance in the amount of activities they participate in

#### Relationships Are Affected

On the one hand, starting new relationships is harder, partly because it is more difficult to meet new people since the participants do not go out a lot any more. On the other hand, participants who use a wheelchair feel like people are less interested in coming to talk with them.

“*You have become asocial, not because you want to, but you just become like that. Whereas in the past when I came to a place where I did know everyone, I could always have a chat with someone”… (P10 – F - 33)*

The reactions from strangers can be hard to deal with. Quite often they do not understand what is going on. Mostly, the environment is not adjusted for people in a wheelchair.

“*I do understand that it is difficult for some people to understand, because… if you are in a wheelchair, people assume that you are in it constantly. And if one moment I get out, I get reactions like why does she need a wheelchair?” (P1 – F - 21)*

Upon becoming sick and receiving the diagnosis, people feel as if their social network decreases. They are invited out less, because the others think that the person probably will say no (again) or will not be able to do what they have planned. It is noticed that acquaintances disappear, but real friends stay. Social media is an important medium for keeping in contact.

“*I felt very hurt because of… well you think those are good friends, they are people who are close and that is… You really do notice that the best ones remain. That is a very hard lesson that I had to learn … And the ones that remain, you have forever”. (P2 – F - 54)*

People with hEDS or HSD find relief in contact with fellow sufferers. They want to share frustrations, emotions and pain, but also positive experiences. They can find support, which makes them feel less lonely.

“*The advantage is that, in my group of friends, I have some people who go through the same thing, to whom I can easily tell everything and where you automatically get an understanding. If I say I have ripped a muscle while coughing...” (P11 – F - 32)*

The relationship one has with a partner changes dramatically. It is hard to let the partner completely understand what they are going through and the partner has to go through a process of acceptance. Participants who are single find it hard not to be able to rely on someone.

“*It also has a tremendous impact on his life choices, like for example his part-time job that is being filled with the things to do at home. It would be totally different if I could provide a fulltime wage, so I also limit his personal development”. (P9 – F - 32)*

Intimate relationships can no longer be taken for granted. The pain and fatigue often influence pleasure. This often means that sex is less spontaneous and sometimes less romantic. During sex, it is possible that they dislocate joints, so they have to find positions to make sure that it is less stressful for the body.

“*But sex is no longer the same, because then you are in a lot of pain and there are things that you try to ban. (…) The doctor once suggested taking a pill half an hour or an hour before we have sex, but then you have to plan everything”. (P2 – F - 54)*

The relationship the participants have with their children depends on the age of the children. All the participants want to give their children a normal childhood and go beyond their own limits. However, they find it frustrating when they have to say that there is something they cannot do.

“*For example, fooling around in the garden with my son is not an option. And for a hug, then I will tell him to come and I'll make sure that I'm sitting comfortably so I can hold him. When he was little, I couldn't take him on my lap”. (P5 – F - 35)*

#### Requesting Help and the Process of Acceptance

Asking for help is very difficult. The participants want to be as independent as possible. Others already help them so much, so it is hard to keep on asking for more help.

“*Whether asking for help is difficult, varies. In my familiar environment it's okay. With strangers, it is difficult. I try to organise my life in a way that it means it is unnecessary to ask for help”. (P9 – F - 32)*

Accepting help is easier when they really know they need it. However, there is always a feeling of guilt because they have to rely on others. Most of the time, they want to spare others, at the expense of themselves.

“*I am a fighter and an independent person. And then suddenly you have to let someone wash you. You almost feel inferior (…) That's one of the most difficult things, asking for help and accepting it. I actually find it more difficult than the pain”. (P8 – F - 42)*

Most of the participants have paid household help. Even though it still can be hard to accept this, it is easier because many people without hEDS/HSD also get help. Moreover, because they get help, they have more energy left to do the things they want to do or like doing.

“*And I have to say, since I have had a cleaning lady, there is a lot more time and energy left”. (P6 – F – 36)*

The participants found it very valuable to still being able to mean something to others. It is comforting for themselves to help others.

*I also have a job where I help people, I get a lot of satisfaction from that”. (P7 – M - 35)*

#### Sense of Identity Is Influenced by the Level of Participation in Society

The number of activities people participate in decreases. A lot of activities are physically too hard to accomplish and people get frustrated when there is something they cannot do any more. Doing the domestic chores becomes difficult and people have to start working less or even to stop working. Hobbies become very limited and people miss the activities they used to do. They find it hard to have lost their identity.

“*It's very difficult to go from one day to the next (…) In fact, over time, you can't do your hobbies anymore and you lose your identity. I don't know what to do with my time”. (P6 – F - 36)*

Many activities are adjusted according to the capabilities. Some people divide activities into smaller ones or structure them more, others just take more time to carry out activities. They try to be inventive and find other solutions so as to be able to do their activities.

“*Like when we perform with the choir, well the others stand up straight and I sit on a barstool. They come on stage from behind and I just come from the side. That way, I have to take only a few steps”. (P3 – F - 47)*

Using assistive devices in public is a barrier, but they still have some energy left to do other things. In using, for example, a wheelchair, it becomes easier to go along with people, rather than staying home alone.

“*I did not want to have an electric wheelchair so quickly, but my shoulders dislocate. You will use that electric wheelchair outdoors, without caring what others think about it, but at least you can… You go from A to B”. (P4 – F - 51)*

Mobility becomes limited, which means that freedom is limited. People find it hard to drive a car, since their joints can dislocate. Taking public transportation is very hard since it is not adapted to people with a disability. An e-bike can help some people to join others when going out.

“*I sometimes let a bus pass me by because there are too many people and I just know that my shoulder will be dislocated by someone pushing me. If I can't sit down, there's a good chance that something will happen”. (P11 – F - 32)*

#### The Search for Life-Balance

Most of the participants define themselves as go-getters. It is hard to define their own limits and they often go beyond them. Persons often feel as if they are destroying their bodies but keep searching what they can do. Peer pressure is experienced as a big challenge in indicating when they have reached their limits.

“*In the beginning I easily went beyond my limits. Wanting to help someone, but actually doing more harm than good for yourself. But gradually, I have learned very well what my limits are”. (P7 – M - 35)*

Activities should be planned carefully but being able to be flexible is still necessary. A balance must be found. It can be hard to be impulsive and most of the time rest should be inserted before and after an activity.

“*Something typical for EDS is the ‘spoon theory'. It says that you get up with 10 spoons that day, but with everything that you do, you lose one spoon. So, you get up in the morning and it costs a spoon, you take a shower, and it costs two spoons. And when there are no more spoons, then you're also done for the day. So, you must keep in mind that you plan your day very well”. (P10 – F - 33)*

An important aspect in finding this balance is to consider the amount of pleasure and the consequences of an activity. Sometimes the participants go beyond their limits intentionally because they feel like a certain activity is too important not to do.

“*What plays a role now is, if I really want to do something, I will consider if it's worth it. Is it worth the effort and having a lot of trouble afterwards? This interview is already going beyond my limits. Sitting here two hours with you that will be very hard for me, but I think it's worth it. The days before and after today are crossed out in my diary”. (P6 – F - 36)*

### Phase 3: Comprehensive Understanding

The findings suggest that having hEDS or HSD influences the activities people have, the way they perform those activities and the amount of activities they participate in. The fact that people with hEDS/HSD have fewer activities indirectly influences their interpersonal interactions and relationships. Firstly, because the participants indicated that they do not go out that much, so it is harder to get to know new people and to keep in contact with their acquaintances. This changes their social network. Secondly, because there are some things the participants cannot do any more, they have to rely on others.

**Figure 1:**
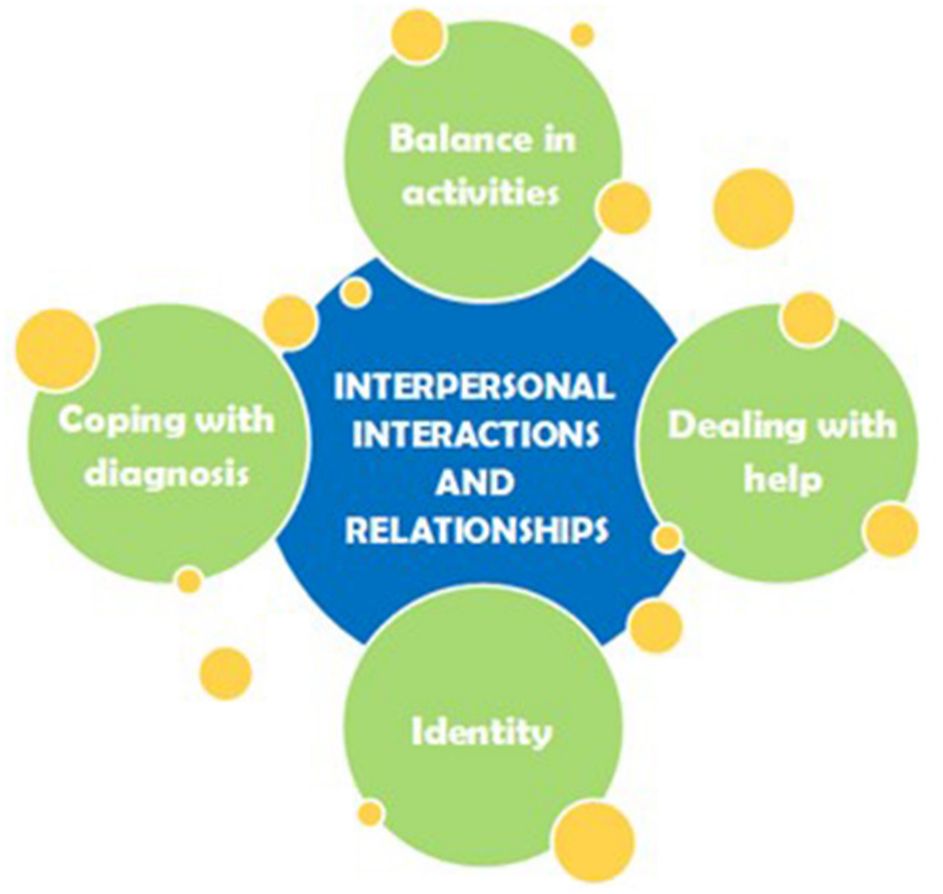
The yellow circles show the aspects influencing interpersonal interactions and relationships that are shown.

It can be said that the interpersonal interactions and relationships of the participants are influenced by their way of coping, the identity that is affected by the current limitations, the balance in the amount of activities and the way of dealing with help. A schematic overview of this comprehensive understanding can be found in Figure 1.

## Discussion

This study shows that the participants are hampered in their social participation. Since social needs are a major concern for people living with a long-term disabling conditions (35), and since the approach to people with hEDS or HSD should be holistic (21), it has been shown to be valuable to explore how patients experience the influence of their condition on interpersonal interactions and relationships. In using a phenomenological hermeneutic approach, the narratives of the participants provided abundant results. There is also the fact that, despite the purposive sampling, the researchers had no influence on the number of participants per gender, since applying for the study was voluntary. Only one male participant who signed up met the criteria. Also, only one participant with HSD was included while all the others have an hEDS diagnosis. The one participant with HSD is the only one who received an updated diagnosis based on the new nosology and did not meet the new hEDS criteria (21).

Getting the diagnosis also affects the self-esteem and self-worth of the participants. Pietromonaco and Collins (23) have shown that self-worth, self-efficacy and perceived control can buffer stress and foster adaptive coping. This is especially important in the context of illness and disease, when symptoms damage self-image and self-esteem. Therefore, it is implied that it is important for patients to be aware of their own self-esteem. The invisibility of the condition has a major impact on this aspect. Another aspect that influences self-esteem and self-worth is the fact that the number of activities persons participate in decreases and the activities themselves change. In 2018, Jespersen et al. found that close connections with family and friends affect quality of life both positively and negatively (22). The fact that support from family and friends is important is corroborated by Murray et al. (12). Both establishing and maintaining social relations play an important role in improving one's social participation (22). The results in this study show that people with hEDS/HSD find it hard to establish social relations and that their social network changes. Furthermore, the relationship with their partner changes. Partners of sufferers from the condition should be understanding (36) and open-minded. It is imperative to make sure that the line between being a partner and being a caregiver is not crossed. The relationship is made even more difficult because having sex is no longer a given.

In following the suggestion of Pietromonaco and Collins (23) that it is important to extend research beyond romantic relationships, this study not only focused on the relationship with a partner, but also on the relationship with friends, children, fellow-sufferers, strangers, health care professionals. The relationship with the broader family could have been considered more in this study, however, other studies have already shown the importance of the support of family (12, 36, 37). Another bias could be that by recruiting the participants through the Flemish Association for Ehlers-Danlos Syndromes and being voluntary, only people that already are more social applied.

An attempt was made to show the fact that a lot of research should still be done on the subject, by adding the yellow circles to the aspects influencing interpersonal interactions and relationships that are shown in Figure 1. Some ideas on future research are listed, such as interviewing the partners and social environment of people suffering from hEDS/HSD to get insight into experiences about living with or being friends with someone who suffers from the condition. Another idea is to conduct a quantitative research, for example with a checklist, on how relationships are experienced by the target group, to see the complete picture and not only be exploratory.

Having hEDS/HSD has an impact on interpersonal interactions and relationships. Support from others is necessary in coping with the diagnosis and others should be open-minded and understanding. Interpersonal interactions and relationships are influenced by the identity, the way people cope with their diagnosis and how they deal with asking for, accepting and giving help. Not only does the social network change, the relationships people have change as well. Different types of relationships are influenced by the disease, including the relationship with their partner, their children, their friends, strangers, fellow-sufferers and health care professionals.

## Data Availability Statement

The datasets presented in this article are not readily available because this is a qualitative research project, based on in-depth, semi-structured interviews with the participants. Publishing the dataset publicly can lead to issues with anonymity. Requests to access the datasets should be directed to stijn.debaets@ugent.be.

## Ethics Statement

The studies involving human participants were reviewed and approved by Ghent University and Ghent University Hospital - Ethical Commission - Ghent - Belgium. The patients/participants provided their written informed consent to participate in this study.

## Author Contributions

SD and MD: conducted the research and writing of the manuscript. PC, FM, and GuV: senior researcher, providing advice regarding the research process, and also providing feedback on the manuscript. GeV: senior researcher and providing advice regarding the research process. DV: senior researcher, providing advice regarding the research process, also providing feedback on the manuscript, and promotor of the research project. All authors contributed to the article and approved the submitted version.

## Conflict of Interest

The authors declare that the research was conducted in the absence of any commercial or financial relationships that could be construed as a potential conflict of interest.

## Publisher's Note

All claims expressed in this article are solely those of the authors and do not necessarily represent those of their affiliated organizations, or those of the publisher, the editors and the reviewers. Any product that may be evaluated in this article, or claim that may be made by its manufacturer, is not guaranteed or endorsed by the publisher.

## Supplementary Material

Supplementary Table 1 SRQR checklist for qualitative research.
