# Supplementary material for: The Impact of Hypermobile “Ehlers-Danlos Syndrome” and Hypermobile Spectrum Disorder on Interpersonal Interactions and Relationships
Source: Front Rehabil Sci. 2022 Apr 11;3:832806. doi: 10.3389/fresc.2022.832806 (PMC9397711; doi:10.3389/fresc.2022.832806)
Supplement: Supplementary Table 1 — SRQR checklist for qualitative research. [file Table_1.DOCX]

| **No.** | **Topic** | **Item checklist** | |
| --- | --- | --- | --- |
| **Title and abstract** | | |  |
| S1 | Title | The title contains a concise description of the nature and topic of the study as qualitative (lived experiences) | |
| S2 | Abstract | The abstract contains a summary of the key elements out of the manuscript. | |
| **Introduction** | | |  |
| S3 | Problem formulation | The significance of the problem has been described in this paragraph, an empirical background has been described. | |
| S4 | Purpose of research methods | The research questions and specific objectives of the project have been described within this manuscript. | |
| **Methods** | | |  |
| S5 | Qualitative approach and research paradigm | The type of qualitative research has been clearly described, next to the rationale behind it. | |
| S6 | Researcher characteristics and reflexivity | The relevant characteristics of the researchers have been described. | |
| S7 | Context | The setting of data gathering has been described, since this could be relevant for this research project. | |
| S8 | Sampling strategy | The sampling strategy has been described extensively | |
| S9 | Ethical issues pertaining to human objects | Ethical considerations have been described, next to the approval of the ethical committee of the University of Ghent. | |
| S10 | Data collection methods | The methods of data collection have been explained in this manuscript. | |
| S11 | Data collection instruments and technologies | The methods of data collection have been explained in this manuscript. | |
| S12 | Units of study | The characteristics of the study population have been described in the text and in an additional table. | |
| S13 | Data processing | The data processing pathway has been described in detail. | |
| S14 | Data analysis | The data processing pathway has been described in detail. | |
| S15 | Techniques to enhance trustworthiness | These techniques have been described. | |
| **Results** | | |  |
| S16 | Synthesis and interpretation | The main finding are clearly explained in the results section of the manuscript. | |
| S17 | Links to empirical data | The evidence is clearly explained, and the overview can be found in the added tables. | |
| **Discussion** | | |  |
| S18 | Integration with prior work, implications, transferability, and contribution(s) to the field | This research project is a part of a bigger research line concerning biopsychosocial approaches of persons with EDS and HSD. | |
| S19 | Limitations | There is a description of the limitations of this study described in the discussion paragraph | |
| **Other** | | |  |
| S20 | Conflicts of interest | There are no known conflicts of interest. | |
| S21 | Funding | There is specific funding within this research project. | |
